# Probing the operability regime of an engineered ribocomputing unit in terms of dynamic range maintenance with extracellular changes and time

**DOI:** 10.1186/s13036-020-00234-5

**Published:** 2020-03-26

**Authors:** Roser Montagud-Martínez, Jordi Ventura, Rafael Ballesteros-Garrido, Arantxa Rosado, Guillermo Rodrigo

**Affiliations:** 1 Institute for Integrative Systems Biology (I2SysBio), CSIC – University of Valencia, 46980 Paterna, Valencia Spain; 2 Present address: Department of Organic Chemistry, University of Valencia, 46100 Burjassot, Valencia Spain

**Keywords:** Biological computation, Regulatory RNA, Synthetic biology, Systems biology

## Abstract

Synthetic biology aims at engineering gene regulatory circuits to end with cells (re)programmed on purpose to implement novel functions or discover natural behaviors. However, one overlooked question is whether the resulting circuits perform as intended in variety of environments or with time. Here, we considered a recently engineered genetic system that allows programming the cell to work as a minimal computer (arithmetic logic unit) in order to analyze its operability regime. This system involves transcriptional and post-transcriptional regulations. In particular, we studied the analog behavior of the system, the effect of physicochemical changes in the environment, the impact on cell growth rate of the heterologous expression, and the ability to maintain the arithmetic functioning over time. Conclusively, our results suggest 1) that there are wide input concentration ranges that the system can correctly process, the resulting outputs being predictable with a simple mathematical model, 2) that the engineered circuitry is quite sensitive to temperature effects, 3) that the expression of heterologous small RNAs is costly for the cell, not only of heterologous proteins, and 4) that a proper genetic reorganization of the system to reduce the amount of heterologous DNA in the cell can improve its evolutionary stability.

## Introduction

The design and subsequent implementation of synthetic gene circuits provide valuable information about the corresponding counterparts found in nature [1]. However, the design process irremediably considers a simplified scenario for tractable purposes. In particular, the circuit design process often assumes a Boolean scenario of input activity (i.e., inducer present or absent in the medium) [2, 3]. Yet, for a comprehensive understanding of the regulatory mechanisms, designer circuits should be analyzed in analog terms in order to recognize the ability of the system to work with ranges of concentrations and hence buffer eventual perturbations in the input signals [4]. This is especially relevant for circuits that operate in non-saturated conditions (e.g., as a result of weak binding constants), as it is the case e.g. of riboregulatory systems [5].

To what extent the design principles exploited to engineer synthetic gene circuits [1] are not influenced by more complex processes (linked to the physicochemical properties of the different biological species and the intricate regulatory circuitry of the cell) is also not entirely known [6], although there are ongoing efforts in this direction [7]. For example, intra- or intermolecular RNA interactions are quite sensitive to changes in temperature, pH, osmolarity, or metal ions [8, 9]. This makes RNA circuits to be eventually sensitive to changes in the medium. On the one hand, because these changes may affect the expression of the RNAs and, on the other hand, because they may affect the interaction mode between them. Notwithstanding, such sensitivity can be exploited to design biosensors, as it is the case of synthetic thermometers based on riboswitches [10].

In addition to variable performance in different environmental scenarios, the engineered circuits will also evolve with the time course (i.e., they will acquire mutations), eventually leading to changes in functionality. These changes can in some cases be drastic, abolishing completely the intended function [11], especially when the heterologous expression causes high burden in the cell [12]. To this end, it is important to evaluate the evolutionary stability of the circuits. Some works have been carried out to study the stability of circuits based on transcription regulation [13, 14], but little is known about how circuits based on regulatory RNAs evolve.

**Fig. 1:**
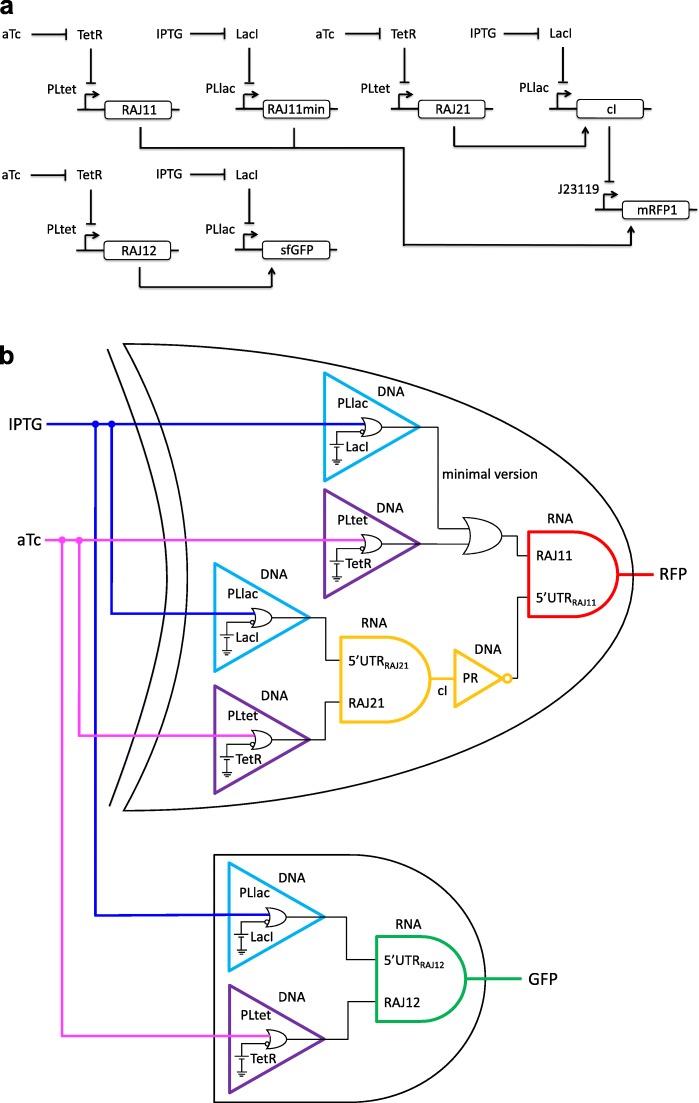
Schematics of the engineered gene circuit implementing a half adder in bacteria **a** Symbolic and **b** logic representation of the circuit. IPTG and aTc are the two molecules that work as input signals in the system (note that the PLlac or PLtet promoter implements an IMPLY gate), while the expressions of an RFP (XOR gate; from plasmid pRHA40) and a GFP (AND gate; from plasmid pRHA12) constitute the output responses

In this work, we considered a recently engineered gene circuit working like a half adder (the combination of XOR and AND gates) [15], which is based on three transcription factors (LacI, TetR, and cI) and four riboregulators (RAJ11, RAJ11min, RAJ12, and RAJ21), illustrated in Fig. 1a. The engineered unit exploits the allosteric ability of LacI and TetR for input sensing and the riboregulators together with cI for implementing the regulatory core. In electronic terms, the XOR gate allows computing the binary sum of the two inputs (output ON when only one input is present, whatever the input, otherwise OFF), while the AND gate allows getting the carry of that sum (output ON only when both inputs are present, otherwise OFF; Fig. 1b) [16]. In the following, we present experimental results that served us to critically analyze the operability regime of this synthetic circuit, which is of importance on the light of the considerable growth of RNA synthetic biology in recent years [17].

## Results

### Analog behavior of the system

**Fig. 2:**
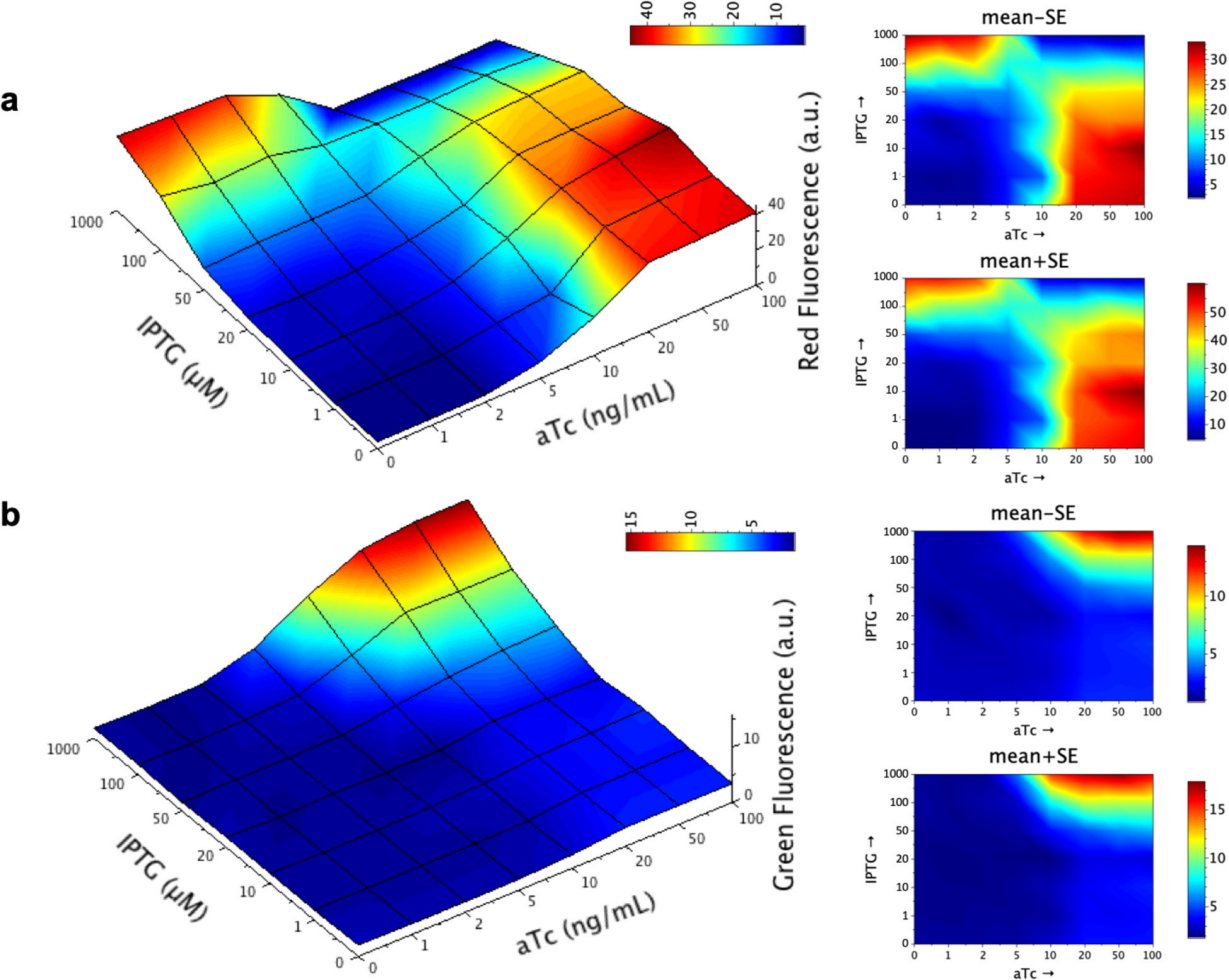
Dynamic range of the engineered gene circuit (*E. coli* co-transformed with plasmids pRHA40 and pRHA12). Fluorescence-based surface response with respect to IPTG and aTc (56 induction conditions tested); mean of four biological replicates. **a** Mean normalized red fluorescence (AND gate). **b** Mean normalized green fluorescence (XOR gate). 3D grid constructed from experimental data, colored by interpolation. On the right, 2D plots showing mean plus/minus standard error

**Fig. 3:**
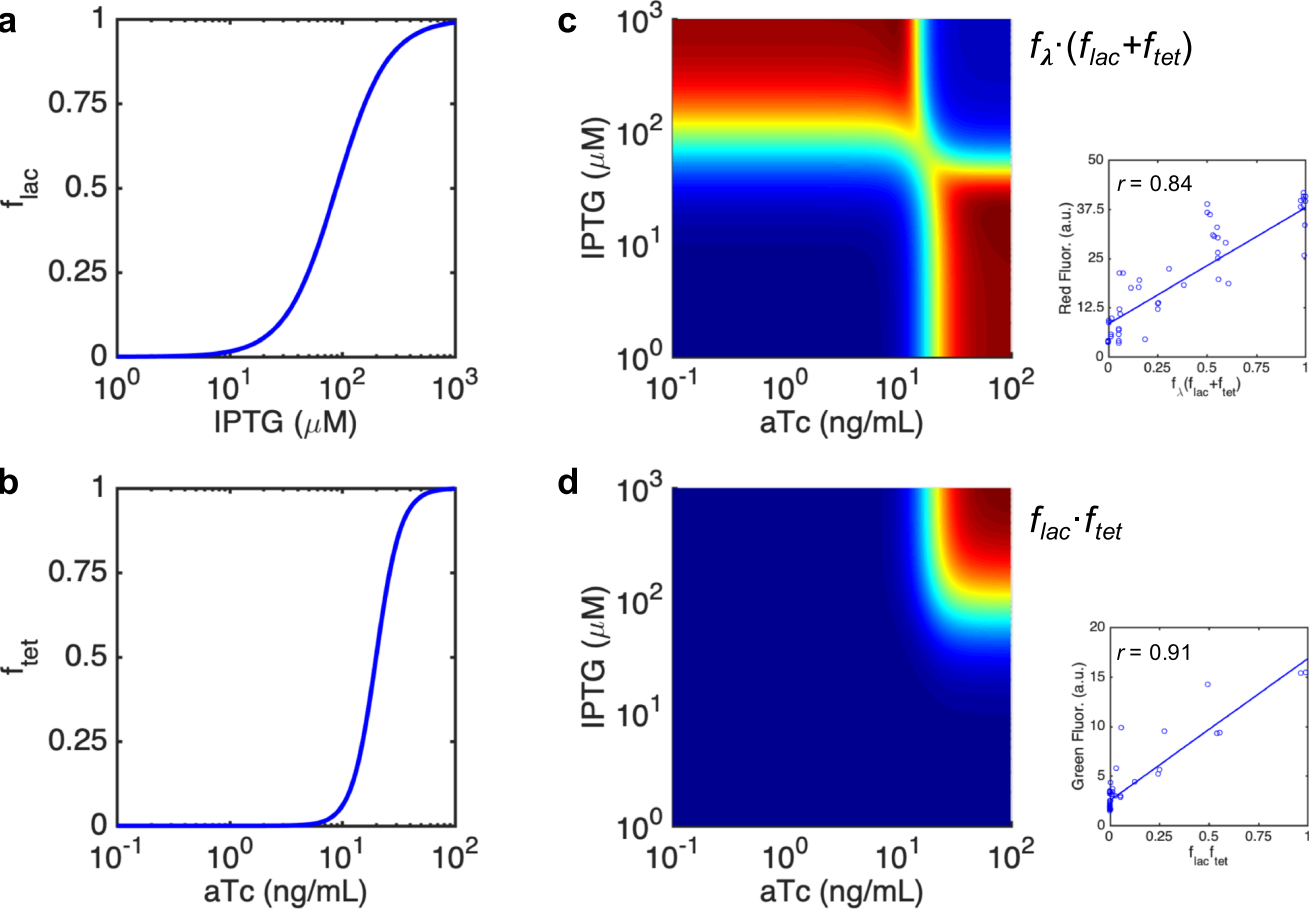
Output response prediction from individual promoter characterizations. **a, b** Dose-response curves of the *lac* and *tet* promoters with respect to IPTG and aTc. **c, d** Prediction of the surface responses of the engineered gene circuit by considering separation of variables. The subplots show the correlation between the predicted values and mean normalized fluorescence data

We analyzed the double output response of the RNA circuit for a two-dimensional concentration gradient of its inducers, isopropyl β-D-1-thiogalactopyranoside (IPTG), ranging from 0 to 1000 μM, and anhydrotetracycline (aTc), from 0 to 100 ng/μL. For that, we monitored the expression of the monomeric red fluorescent protein (mRFP1) and the superfolder green fluorescent protein (sfGFP) for each induction condition (Fig. 2). We observed that the state transitions agree in both output channels (at ~ 100 μM IPTG and at ~ 10 ng/mL aTc). In essence, this is because synthetic riboregulators act in a linear regime; that is, protein expression scales with the product between the messenger RNA (mRNA) and small RNA (sRNA) concentrations [18]. Then, the non-linearity of the response comes from the transcriptional regulation with engineered promoters [19], which are basically the same for the XOR and the AND gates (despite some modifications in the operators). This entails a separation of input variables to describe the output response [20]. Indeed, sfGFP expression can be approached as proportional to the product of the dose-response curves for the *lac* and *tet* promoters, sfGFP ~ *f*_*lac*_·*f*_*tet*_, whilst mRFP1 expression as proportional to the sum of these two dose-response curves times the dose-response curve for the λ promoter, mRFP1 ~ *f*_λ_·(*f*_*lac*_ + *f*_*tet*_), as shown in Fig. 3. Linear correlations between experimental and theoretical values, the latter computed with the aforementioned algebraic equations for the different IPTG and aTc concentrations, revealed a good agreement (Pearson correlation coefficient *r* > 0.80 and *P*-value < 10^− 10^ in both cases).

Moreover, this scheme entails that the system is able to correctly process two concentration ranges instead of two concentration values. For instance, values of IPTG up to 20 μM would correspond to zero in binary code or values of aTc from 40 to 100 ng/mL to one in binary code too. Basically, this allows being tolerant to fluctuations occurring at the input level from extracellular processes that are extrinsic to the circuit, such as molecular titration or degradation by environmental agents.

### Environmental robustness of the system

**Fig. 4:**
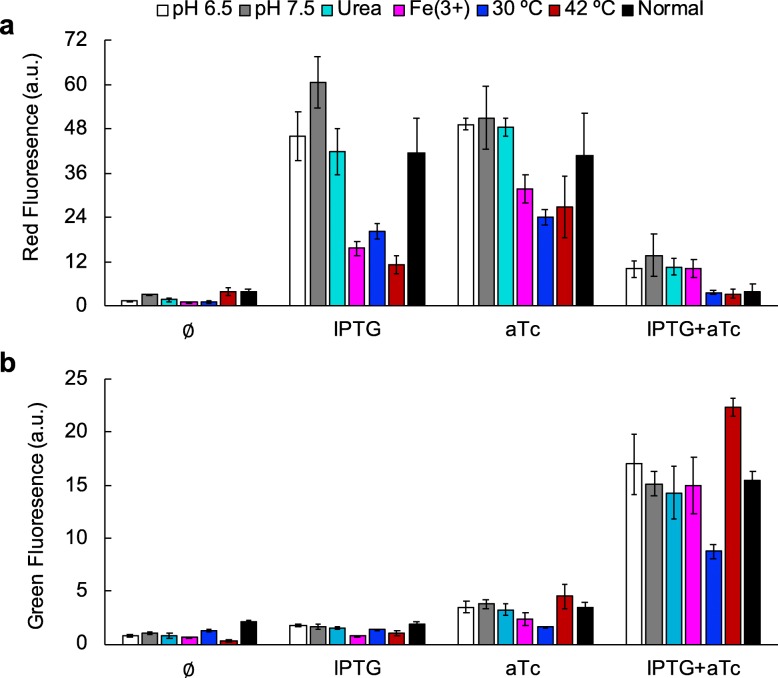
Environmental robustness assessment of the engineered gene circuit (*E. coli* co-transformed with plasmids pRHA40 and pRHA12). Fluorescence monitoring for each induction condition (IPTG, aTc) when the culture medium changes (pH variation, urea addition, Fe^3+^ addition, and temperature variation). **a** Normalized red fluorescence. **b** Normalized green fluorescence. Error bars correspond to standard errors over four biological replicates

To study how different environmental conditions affect the performance of the RNA circuit, we decided to vary temperature and pH. We also studied the effect of meaningful amounts of heavy metals that are essential for life, such as Fe^3+^, Cu^2+^, and Zn^2+^, as well as of urea, a denaturing agent of RNAs and proteins. However, bacteria did not tolerate the presence the Cu^2+^ or Zn^2+^ in the medium. Upon characterizing the circuit in all possible environments for each induction condition (Fig. 4), we first observed certain robustness to fluctuations in pH in both output channels. We only noted certain impact on the ability of the transcription factor cI to repress mRFP1. The addition of urea neither left a significant imprint, but Fe^3+^ caused a reduction in red fluorescence upon induction with IPTG or aTc, although not in green fluorescence. Arguably, high amounts of Fe^3+^ might quench the fluorescence from mRFP1 [21]. Conversely, we found both output channels sensitive to changes in temperature. We hypothesized that the higher temperature, the greater the transcription rates of the genes (the expression of a control sfGFP with an active ribosome-binding site and under the *lac* promoter confirms this trend; Fig S1) and also the greater the chances to unfold a structured 5′ untranslated region and then produce an expression leakage, especially in plasmids of high-copy number. For mRFP1, nonetheless, the effect is not as straightforward as for sfGFP. An expression leakage of cI at high temperature can explain the reduction in mRFP1 expression upon induction with IPTG or aTc.

**Fig. 5:**
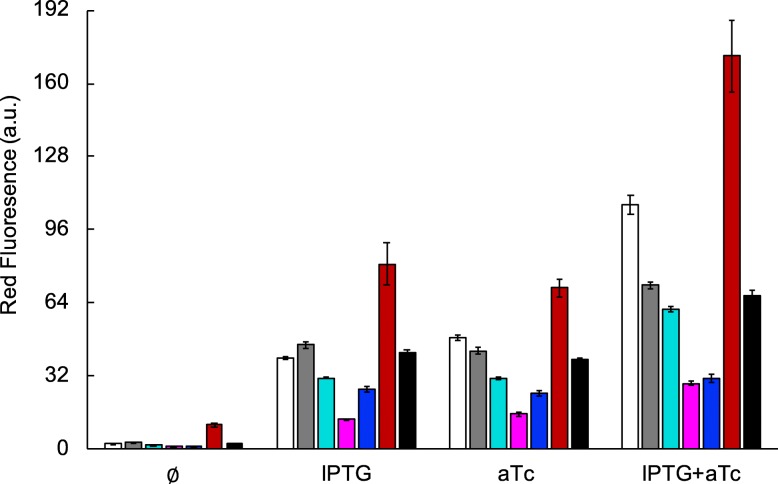
Environmental robustness assessment of a control system implementing an OR gate (*E. coli* transformed with plasmid pRHA37; this system lacks the cI repressor so that mRFP1 is only regulated post-transcriptionally). Fluorescence monitoring (normalized red fluorescence) for each induction condition (IPTG, aTc) when the culture medium changes (pH variation, urea addition, Fe^3+^ addition, and temperature variation; same legend as in Fig. 4). Error bars correspond to standard errors over four biological replicates. Color legend as in Fig. 4

To confirm the proposed mode of action of temperature, we considered a previously-constructed genetic variant of the system of study in which there is no cI-mediated repression of mRFP1, and then the reporter is only regulated post-transcriptionally. Note that this circuit works like an OR gate [15]. Fluorescence readouts now revealed the monotonous increase of mRFP1 expression with temperature in all induction conditions (Fig. 5). We also observed a fluorescence boost with both inducers at low pH, but we attributed this to a cell growth defect of this system in this condition. Indeed, as absolute fluorescence is normalized by absorbance to get an estimate of expression, some stressing conditions for the cell can lead to such boosts as a result of low growths.

### Genetic burden of the system

**Fig. 6:**
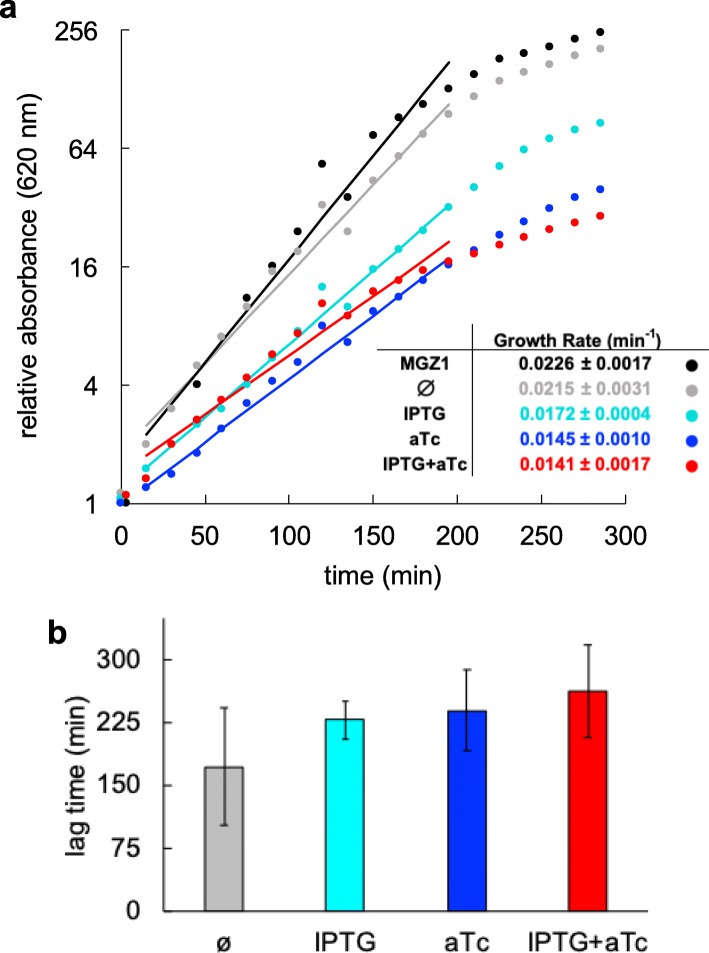
Genetic burden of the engineered gene circuit (*E. coli* co-transformed with plasmids pRHA40 and pRHA12). **a** Relative absorbance with time for each induction condition (IPTG, aTc; data for a representative replicate). Lines correspond to exponential fittings. The inset shows the growth rate values (mean and standard deviation) from four biological replicates. **b** Lag time before exponential growth for each induction condition (IPTG, aTc) from diluted overnight cultures. Error bars correspond to standard deviations from four biological replicates

To assess the impact of the synthetic circuit on the chassis cell, we quantified the growth rate in exponential phase for each induction condition [22]. As expected, we found that as long as the heterologous species were expressed (sRNAs and proteins), cells grew slower (Fig. 6a). In fact, cells grew on average a 34% slower with both inducers than without them. But we know that *i*) in absence of inducers, negligible amounts of sRNAs or proteins are produced; *ii*) when IPTG is present, the system only expresses one sRNA (RAJ11min) and one protein (mRFP1); *iii*) when aTc is in the medium, the system expresses three sRNAs (RAJ11, RAJ12, and RAJ21) and one protein (mRFP1); and *iv*) in the presence of both inducers, there are four sRNAs (RAJ11, RAJ11min, RAJ12, and RAJ21) and two proteins (sfGFP and cI) expressed. A statistical analysis by grouping the conditions as non-induced (gray line in Fig. 6a), induction with IPTG alone (cyan line), and induction with aTc (either alone or in conjunction with IPTG; blue and red lines) resulted in the more significant difference (one-way ANOVA, df 2, *P*-value = 3·10^− 5^); more than controlling for the number of expressed proteins.

In addition, we quantified the time required for the cells to enter in exponential phase from diluted cultures (lag time) for each induction condition [20]. We found that the lag time increased on average about 1 h when there was only one inducer in the medium, whilst it was about 1.5 h when the two inducers were present (Fig. 6b). Yet, statistical analyses only revealed a significant difference when controlling for induction (data points from cyan, blue, and red bars combined) vs. non-induction (gray bar; one-way ANOVA, df 1, *P*-value = 0.026), although with a narrow margin. Taken together, our results revealed that the expression of heterologous sRNAs can be as costly for the cell as the expression of heterologous proteins. This challenges a conventional wisdom in which ribosome allocation mainly limits cell growth rate [12].

### Evolutionary stability of the system

**Fig. 7:**
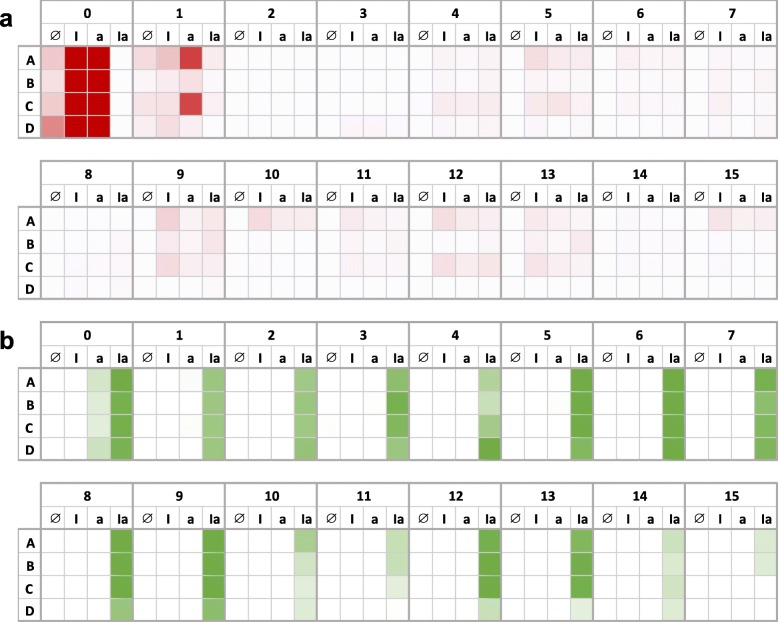
Evolutionary stability of the engineered gene circuit (*E. coli* co-transformed with plasmids pRHA40 and pRHA12). Continuous fluorescence monitoring of four biological replicates (A-D) over 2 weeks for each induction condition (I, IPTG; a, aTc). **a** Normalized red fluorescence. **b** Normalized green fluorescence

To evaluate the evolutionary stability of the system (i.e., its ability to behave as designed with time), we set up an experiment of serial dilutions by carrying in parallel different bacterial populations [23]. To simulate a changing environment, the induction condition was varied with time (none in days 0 and 1, IPTG in day 2, aTc in day 3, IPTG plus aTc in day 4, none again in day 5, and so on). Every day, circuit functionality was assessed by fluorometry. Surprisingly, we found that mRFP1 expression was lost in all lines in just one passage (corresponding to 6.64 generations), whilst sfGFP was able to hold for ten passages (Fig. 7). We attributed these courses to the fact that mRFP1 was expressed from a very high-copy number plasmid (pUC ori), whilst the plasmid from which sfGFP was expressed presents a more moderate copy number (E93K mutated pSC101 ori). Sequencing results of the XOR gate after the second passage revealed a mix of sequences in all lines (i.e., two or more nucleotide peaks at the same location). This was throughout the genetic cassette, including the regulatory and mRFP1 coding regions, then suggesting the accumulation (but not yet fixation) of deleterious mutations. By contrast, we found fixed mutations in the sfGFP coding region in all lines (nucleotide deletions that disrupt the reading frame, at different points in each line), but not in the regulatory region, by sequencing the AND gate at last day of the evolutionary experiment. Using a plasmid of high-copy number in which sfGFP is expressed under the *lac* promoter, we also found that after few passages the fluorescence was lost (Fig. S2), which supports the idea that the instability comes more from the backbone than from the circuit itself.

**Fig. 8:**
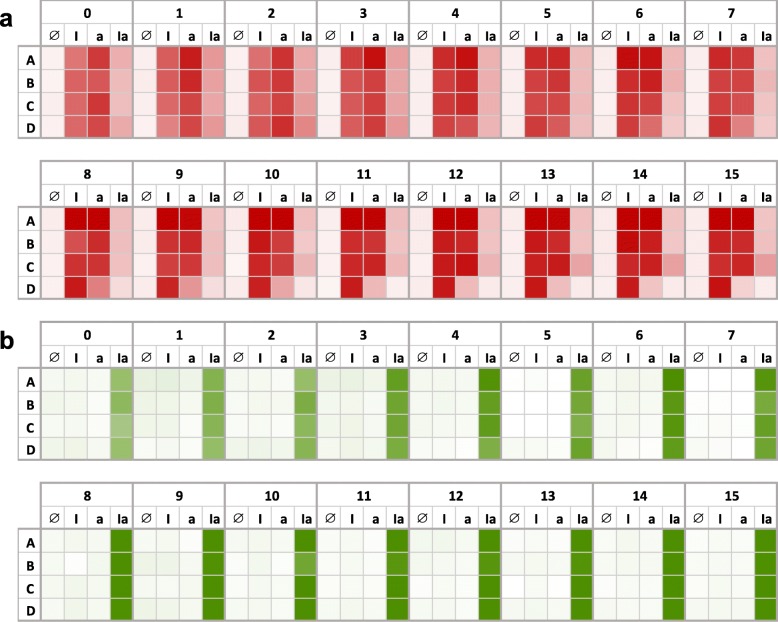
Evolutionary stability of the engineered gene circuit when it is deployed from a single plasmid (*E. coli* transformed with plasmid pRHA1240). Continuous fluorescence monitoring of four biological replicates (A-D) over 2 weeks for each induction condition (I, IPTG; a, aTc). **a** Normalized red fluorescence. **b** Normalized green fluorescence

With the aim of increasing the evolutionary stability of the system, we placed the genetic cassette corresponding to the XOR gate into the plasmid carrying the AND gate (Fig. S3; thereby reducing substantially the amount of heterologous DNA in the cell). Quantitatively, we observed lower red fluorescence upon induction with IPTG or aTc and a marked leakage with the two inducers, suggesting that now the repressor cI was not sufficiently expressed as a result of a worse performance of the riboregulatory system RAJ21. Repeating again the experimental evolution, we found that this new system was able to maintain its arithmetic functionality much more time (Fig. 8). In particular, mRFP1 was correctly expressed over the 2 weeks in three lines. Only in line D, the expression dropped after 1 week when inducing with aTc. Sequencing results at last day revealed a 25 bp deletion in the *tet* promoter controlling the expression of the sRNA RAJ11, which is explicative of the observed behavior. In line C, nonetheless, we could not identify a unique sequence from the chromatogram in the region coding for the sRNAs RAJ11 and RAJ21, suggesting that neutral mutations might be accumulated there. Furthermore, sfGFP was correctly expressed over the 2 weeks in all lines, better than before, and sequencing results at last day confirmed the no acquisition of mutations in that cassette.

## Discussion

This work contributes to better understand the operability regime of engineered circuitries based on regulatory RNAs. Notably, our results allow disclosing a series of principles in order to predict the effect of a variety of changes, and in turn develop strategies to mitigate them. First, by exploiting a pre-characterized transcriptional layer for RNA circuit engineering [3], and noting that riboregulatory performance can be correctly predicted from energetic and structural calculations through the use of RNA folding routines [24, 25], the prediction of the output responses for combinatorial induction conditions is possible, as our results point out. We employed a simple mathematical model based on algebraic equations (gene expression in steady state) to anticipate relative fluorescence values from specific input concentrations. Subsequently, we identified temperature effects as environmental variations with clear impact on circuit performance; effects that appear as a consequence of coupling transcriptional and post-transcriptional regulations. In this regard, a circuit implementation with tightly-regulating transcription factors only in the sensory layer and a pure RNA-based actuation layer (i.e., RNA-based rather than cI-based repression in the XOR gate) [26] might be an option to limit, at least in part, the impact of changes in temperature. In addition, our growth curves suggest that the expression of heterologous sRNAs is costly for the cell, as it can be the expression of heterologous proteins [12]. We hypothesized that the overexpression of sRNAs from high-copy number plasmids creates a high demand for the different ribonucleases that is translated into cell growth defects, atop of genome-wide transcriptomic perturbations [27]. Finally, our evolutionary experiments revealed a trade-off for synthetic RNA circuits. On the one hand, these circuits require the expression from high-copy number plasmids (or from T7 promoters [28]) to deal with binding constants at the micromolar scale [5]. On the other hand, these plasmids introduce an elevated cost for the cell and then they are prone to the accumulation of deleterious mutations (or even to the induction of sizable changes in RNA or protein concentration with time that may disrupt function [29]). Perhaps, circuits involving synthetic sRNAs with domains able to recruit endogenous RNA chaperones [30] and integrated into the chromosome would be the best strategy to enhance substantially the evolutionary stability.

## Methods

### Strains, plasmids, and reagents

*E. coli* Dh5α was used for cloning purposes, while *E. coli* MG1655-Z1 (*lacI*^+^, *tetR*^+^) was used as a chassis cell to express our genetic systems (gifted by M.B. Elowitz) [31]. This was co-transformed with plasmids pRHA12 and pRHA40 [15] by electroporation, which implement a genetic half adder. This chassis cell was also transformed with plasmid pRHA27, plasmid pRHA37 [15], or plasmid pRHA1240. This latter plasmid was obtained by digesting pRHA40 with EcoRI and BamHI and inserting the resulting cassette into pRHA12 by digesting with EcoRI and ligating with T4 DNA ligase.

LB medium was used for overnight cultures, while M9 minimal medium (1x M9 salts, 2 mM MgSO_4_, 0.1 mM CaCl_2_, 0.4% glucose, 0.05% casamino acids, and 0.05% thiamine) for characterization cultures. Ampicillin and kanamycin were used as antibiotics at the concentration of 50 μg/mL. IPTG and aTc were used as inducers at the concentrations of 1000 μM and 100 ng/mL, respectively, for maximal induction of the synthetic PL-based promoters [19]. Concentration gradients of inducers were also considered: 0, 1, 10, 20, 50, 100, and 1000 μM IPTG and 0, 1, 2, 5, 10, 20, 50, and 100 ng/μL aTc. Compounds provided by Sigma-Aldrich.

### Preparation of cultures for characterization

Cultures (2 mL) inoculated from single colonies (four replicates) were grown overnight in LB medium at 37 °C and 200 rpm. Cultures were then diluted 1:100 in M9 minimal medium (2 mL) and were grown for 5–7 h at 37 °C and 200 rpm to reach exponential phase (OD_600_ around 0.5). In the case of cultures from the experimental evolution, glycerol stocks were diluted 1:50 in M9 minimal medium in a microplate (200 μL) and were grown for 5–7 h at 37 °C and 1000 rpm to reach exponential phase (OD_600_ around 0.5) in a plate shaker (PST-60HL, Biosan). Cultures were then diluted, in both cases, 1:50 in M9 minimal medium with appropriate inducers (IPTG, aTc).

### Fluorescence quantification

From the prepared cultures for characterization, the microplate (96 wells, black, clear bottom; Corning) was loaded (200 μL/well). The microplate was then incubated in the plate shaker at 37 °C (unless otherwise specified) and 1000 rpm up to 7 h (to reach an OD_600_ around 0.5–0.7). Subsequently, the microplate was assayed in a fluorometer (Varioskan Lux, Thermo Sci.) to measure absorbance (600 nm), green fluorescence (excitation: 485 nm, emission: 535 nm), and red fluorescence (excitation: 570 nm, emission: 610 nm). Mean background values of absorbance and fluorescence, corresponding to M9 minimal medium, were subtracted to correct the signals. Normalized fluorescence was calculated as the ratio of fluorescence and absorbance. The mean value of normalized fluorescence corresponding to non-transformed cells was then subtracted to obtain a final estimate of expression.

### Empirical model to predict the response

The individual dose-response curves of the promoters used to implement our circuit were already characterized. Promoter activity follows the sigmoidal function $f=\frac{1}{1+{\left(\frac{X}{K}\right)}^n}$, where *X* is the concentration of the regulator, *K* the effective dissociation constant, and *n* the Hill coefficient. Then, we have *i*) *K* = 89 μM (relative to IPTG) and *n* = − 1.9 for the *lac* promoter [19], *ii*) *K* = 20 ng/mL (relative to aTc) and *n* = − 4 for the *tet* promoter [19], and *iii*) *K* = 55 nM (relative to cI) and *n* = 2.4 for the λ promoter [32]. Consequently, we can write mRNA_mRFP1_ ~ *f*_λ_, RAJ11min ~ *f*_*lac*_, RAJ11 ~ *f*_*tet*_, mRNA_cI_ ~ *f*_*lac*_, RAJ21 ~ *f*_*tet*_, mRNA_sfGFP_ ~ *f*_*lac*_, and RAJ12 ~ *f*_*tet*_. Of note, *f*_λ_ also depends on IPTG and aTc, as cI ~ *f*_*lac*_·*f*_*tet*_ (for this we assumed a proportionality factor of 250 nM). Finally, it turns out that mRFP1 ~ mRNA_mRFP1_·(RAJ11min + RAJ11) ~ *f*_λ_·(*f*_*lac*_ + *f*_*tet*_) and sfGFP ~ mRNA_sfGFP_·RAJ12 ~ *f*_*lac*_·*f*_*tet*_*.*

### Different environmental conditions

To assess the effect of temperature on the genetic half adder, the microplate was also incubated in the plate shaker at 30 or 42 °C. To assess the effect of pH, the culture medium in the microplate was prepared to be more acid (6.5, with HCl) or more alkaline (7.5, with NaOH). Finally, to assess the effect of life-related compounds that can be present in the environments where bacteria can be deployed, the medium was supplemented with FeCl_3_ (Fe^3+^), Cu(NO_3_)_2_ (Cu^2+^), Zn(NO_3_)_2_ (Zn^2+^), or CO(NH_2_)_2_ (urea), leading to ~ 1 mM in all cases.

### Growth rate curves

From the prepared cultures for characterization, the microplate was loaded (200 μL/well). The microplate was then incubated for 24 h at 37 °C with shaking in a spectrophotometer (Multiskan FC, Thermo Sci.), measuring absorbance (620 nm) every 15 min.

### Experimental evolution

Cultures (2 mL) inoculated from single colonies (four replicates) were grown overnight in LB medium at 37 °C and 200 rpm. Propagation of the lines during 15 d was carried out by imposing a daily 1% bottleneck (dilution 1:100), also in LB at 37 °C and 200 rpm. Each day, the induction condition was varied (from none, to IPTG, to aTc, to IPTG plus aTc) to simulate a changing environment. A daily fossil record of each line was generated by adding glycerol 20% to an equal volume of culture.

### Sequencing of the genetic circuit

To sequence the cassette corresponding to the XOR gate, we used the following primers: CTTTGATAACGTCTTCGGAGGAAGC (forward, landing in the mRFP1 coding region), CACTCCACTCTGCCCCATAC (reverse, landing in the cI coding region), and CCCAGTCACGACGTTGTAAAACG (forward, landing in the backbone upstream of the insert). To sequence the cassette corresponding to the AND gate, we used the following primers: GCTTCTTCCGCTAGCATCGA (forward, landing in the backbone upstream of the insert) and ACAGCTCTTCGCCTTTACGG (reverse, landing in the sfGFP coding region). Sequencing carried out by Eurofins Genomics.

#### ACKOWLEDGEMENTS

We thank the CSIC Open Access publication support initiative through its Unit of Information Resources for Research.

## Supplementary information

**Additional file 1: Fig. S1.** Environmental robustness assessment of a control system (*E. coli* transformed with plasmid pRHA27; this system just expresses sfGFP under the control of the *lac* promoter). Fluorescence monitoring (normalized green fluorescence) when the culture medium changes (pH and temperature variation). Error bars correspond to standard errors over four biological replicates.

**Additional file 2: Fig. S2.** Evolutionary stability of a control system (*E. coli* transformed with plasmid pRHA27). Continuous fluorescence monitoring of four biological replicates (A-D) over one week.

**Additional file 3: Fig. S3.** Schematics of the plasmids pRHA40, pRHA12, and pRHA1240.

**Additional file 4: Dataset S1.** Experimental and theoretical data of the half adder behavior. The Excel file contains an implementation of the mathematical model.

## Data Availability

See Dataset S1 for data. Plasmids pRHA40 and pRHA12 are deposited in Addgene (with refs. #138941 and #138942, respectively). Please, contact the authors for other requests.

## Abbreviations

IPTG: Isopropyl β-D-1-thiogalactopyranoside
aTc: Anhydrotetracycline
mRFP1: Monomeric red fluorescent protein
sfGFP: Superfolder green fluorescent protein
mRNA: Messenger RNA
sRNA: Small RNA
