# Supplementary figures and images for: Probing the operability regime of an engineered ribocomputing unit in terms of dynamic range maintenance with extracellular changes and time
Source: J Biol Eng. 2020 Mar 26;14:12. doi: 10.1186/s13036-020-00234-5 (PMC7098154; doi:10.1186/s13036-020-00234-5)

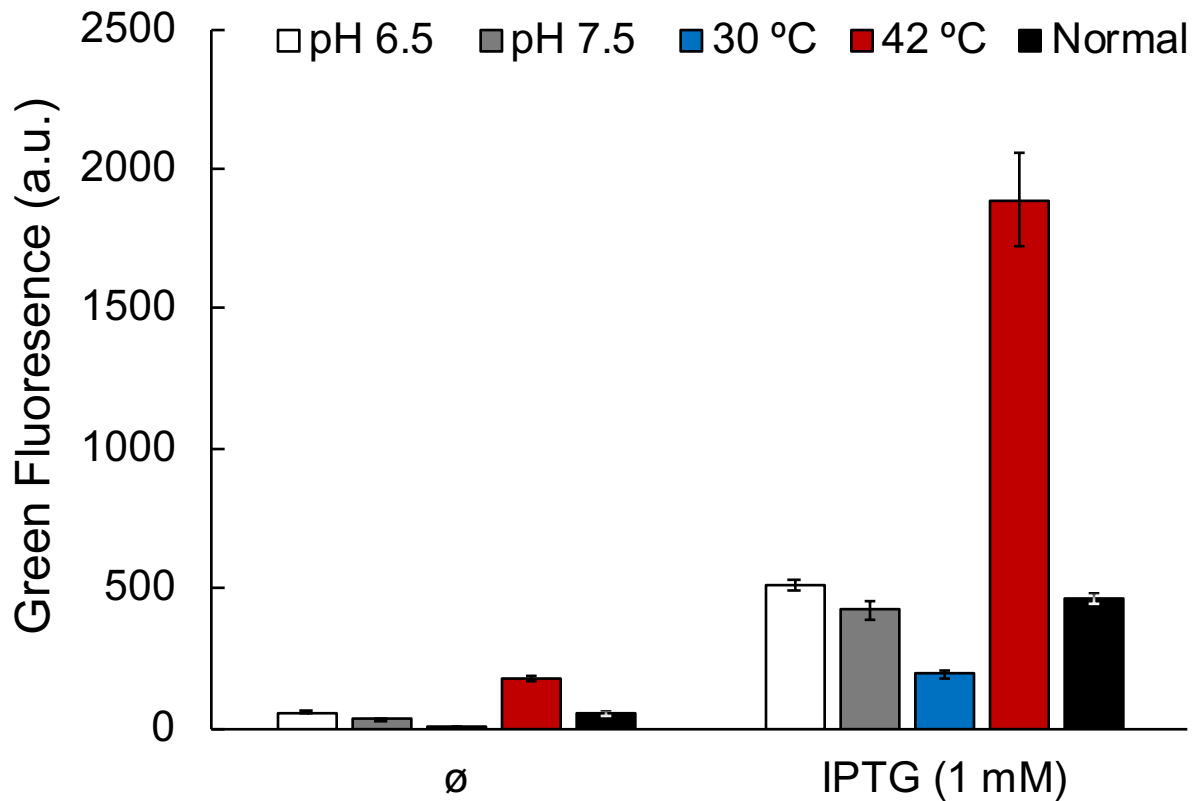

Supplement: Supplementary file 1 — Additional file 1: Fig. S1. Environmental robustness assessment of a control system (E. coli transformed with plasmid pRHA27; this system just expresses sfGFP under the control of the lac promoter). Fluorescence monitoring (normalized green fluorescence) when the culture medium changes (pH and temperature variation). Error bars correspond to standard errors over four biological replicates. [file 13036_2020_234_MOESM1_ESM.pdf]

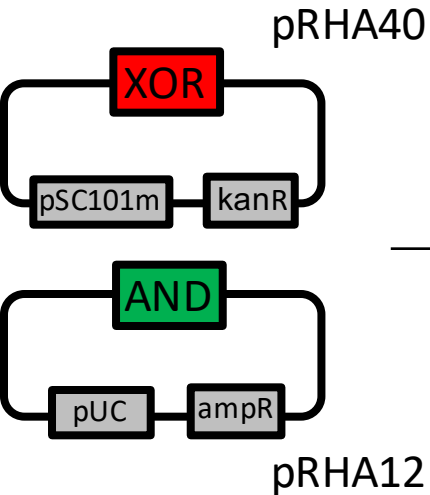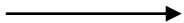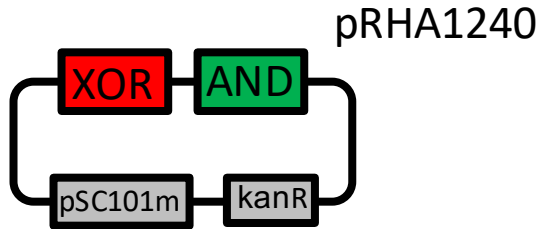

Supplement: Supplementary file 3 — Additional file 3: Fig. S3. Schematics of the plasmids pRHA40, pRHA12, and pRHA1240. [file 13036_2020_234_MOESM3_ESM.pdf]
